# Lack of evidence of viability and infectivity of SARS-CoV-2 in the fecal specimens of COVID-19 patients

**DOI:** 10.3389/fpubh.2022.1030249

**Published:** 2022-10-20

**Authors:** Madhuri Joshi, Sreelekshmy Mohandas, Sharda Prasad, Manohar Shinde, Nutan Chavan, Pragya D. Yadav, Mallika Lavania

**Affiliations:** ^1^Enteric Viruses Group, ICMR-National Institute of Virology, Pune, India; ^2^Microbial Containment Laboratory, ICMR-National Institute of Virology, Pune, India; ^3^Electron Microscopy and Histopathology Group, ICMR-National Institute of Virology, Pune, India

**Keywords:** SARS-CoV-2, COVID-19, fecal, Real time RT-PCR, TEM, NGS

## Abstract

SARS-CoV-2 can be shed in feces and can enter sewage systems. In order to implement effective control measures and identify new channels of transmission, it is essential to identify the presence of infectious virus particles in feces and sewage. In this study, we attempt to utilize Molecular techniques, cell cultures and animal models to find out the infectivity of SARS-CoV-2 in the feces of COVID-19 patients. Our findings exclude the presence of infectious virus particles, suggesting that fecal-oral transmission may not be the main mode of transmission. Larger-scale initiatives are nevertheless required, particularly considering the emergence of new viral strains.

Assessments of the magnitude of ecological contamination by viruses are inside the domain of environmental virology. As we comprehend the genuine significance of viruses, proper control measures can be implemented to lessen the public health hazards. The first instance of Coronavirus disease 2019 (COVID-19) was discovered in China in December 2019, and it has since spread across the globe. COVID-19 cases totaled 563,107,209 worldwide as of July 13, 2022, including 43,672,155 in India (1). As the pandemic situation of COVID-19 worsened, it was suggested that along with the respiratory route alternative channels of transmission, such as fecal-oral and airborne transmission, may exist. For Coronaviruses like SARS-CoV and MERS-CoV, the fecal-oral route was previously accepted as a method of transmission (2–4). The detection of SARS-CoV-2 RNA in anal/rectal swabs (5), stool samples (6–9) and wastewater/sewage (10, 11), along with studies demonstrating a high detection rate and viral levels equivalent to earlier SARS epidemics in fecal samples, of COVID-19 patients has been documented. Shared restrooms were blamed in one study for high rates of familial clustering (12, 13). Similarly, a longer duration of detection of SARS-CoV-2 RNA in feces as compared to respiratory specimens documented (14–16) may possibly suggest that the virus may be actively replicating in the patient's gastrointestinal tract and that fecal–oral transmission might occur after viral clearance in the respiratory tract. In our previous study with a 62% fecal detection rate, no significant difference in mean duration of RNA detection in the GI tract asymptomatic and symptomatic cases of COVID-19 was documented (16). The studies on asymptomatic individuals as infectious during the early stage of infection were reported with rare cases becoming long-term virus carriers and lead to an apparent spread of the virus similar to symptomatic cases (17). The possibility of rare long-term carriers becoming virus reservoirs, with the potential to cause recurrent outbreaks, needs to be studied and will have important implications for future SARS-CoV-2 public health and surveillance, and our understanding of the SARS-CoV-2 virus. In developing countries due to a majority of asymptomatic infections and crowded places, the possibility of the fecal-oral route of transmission of SARS-CoV-2 may be high and prompted us to explore the possibility of viable infectious virions in fecal material.

In the present study [ethically approved by ICMR-NIV Ethics committee: 20-2-2 R], fecal specimens of COVID-19 patients were subjected to RT-PCR, digital RT-PCR, Next Generation sequencing (NGS), isolation in cells and animal models, and electron microscopic studies. Ten fecal specimens of COVID-19 patients (aged between 5 and 70 years) admitted in COVID-19 Care Center in Pune City, Maharashtra state, India during the second wave period [Jun-Aug 2021] were included in the study. Among ten patients, six were experiencing symptoms like fever, cough, sore throat, chest pain, wheezing, abdominal pain, vomiting/nausea, diarrhea, loss of taste and smell, while the remaining four were asymptomatic (Table 1). SARS-CoV-2 strains were detected using E and ORF gene based Real Time Reverse Transcription Polymerase chain reaction (RT-PCR) assays in paired throat and fecal specimens using protocols described earlier (18). The testing of throat swabs and fecal specimens (collected between 0 and 6 post onset day) was performed at the National Influenza Center, NIV, Pune. All throat swab specimens were positive with Ct values between 18–27 and 21–29 for E gene and ORF region respectively and fecal specimens with Ct values 19.5–25.9 and 22.6–28.1 for E gene and ORF region of SARS-CoV-2 respectively (Table 1). The viral RNA load in the fecal specimens was estimated using N gene based digital RT-PCR assay as per manufacturer's instructions (SARS-CoV-2 N1 + N2 Primer Probe Assay Kit, Qiagen, Germany). Thermal cycling was performed at 50°C for 40 min for reverse transcription, followed by 95°C for 2 min and then 40 cycles of 95°C for 05 s, and 60°C for 30 s. Analysis of the viral RNA load in fecal specimens indicated 5526.2–15.2 copies per/μL of RNA.

**Table 1:** Clinical and demographic data of the SARS-CoV-2 positive patients along with real-time RT-PCR, next generation sequencing and electron microscopic analysis of the corresponding fecal specimens.

**Sample ID**	**Age/gender**	**POD**	**Admission signs and symptoms**	**Ct values of real time RT-PCR**				**NGS analysis -total reads**	**Lineage [variant]**	**EM analysis**
				**Throat swab**		**Stool**				
				**E gene**	**ORF gene**	**E gene**	**ORF gene**			
BR-21-195	65/M	3	Fever, cough, lower chest wall indrawing, diarrhea, loss of taste, smell	26	28	19.5	22.7	13857	B.1.617.1 [Kappa]	Negative
BR-21-200	5/F	0	Fever, cough, bloody/ hemoptysis, sore throat, runny nose, abdominal pain, diarrhea, loss of taste, smell	20	22	19.8	23.2	28237	B.1.617.2 [Delta]	Negative
BR-21-238	7/M	3	Asymptomatic	23	25	21	24.7	20314	B.1.617.2 [Delta]	Negative
BR-21-555	24/M	3	Fever, cough, runny nose, altered consciousness/confusion, vomiting/nausea, diarrhea, new loss of taste and smell	21	29	23.3	25.3	27370	B.1.617.2 [Delta]	Negative
BR-21-537	8/M	6	Fever, cough, sore throat, chest pain, wheezing, abdominal pain, vomiting/nausea, loss of taste and smell	18	21	25.9	28.1	27825	B.1.617.2 [Delta]	Negative
BR-21-772	70/M	6	Asymptomatic	25	26	25.9	28.1	27172	B.1.617.2 [Delta]	Distinct coronavirus ++ 55nm core to spike GP+. Intact particles
BR-21-803	28/M	1	Fever, cough	27	28	23.3	25.3	14254	B.1.617.1 [Kappa]	Negative
BR-21-813	32/M	3	Asymptomatic	23	27	23.5	27.1	15536	Unassigned	High background debris of phages. No coronavirus
BR-21-815	14/M	1	Asymptomatic	25	28	21.6	24.4	26393	B.1.617.2 [Delta]	Positive with low frequency coronavirus. 2 VLP in 4 fields
BR-21-820	39/M	2	Fever, cough	25	27	20.1	22.6	17429	B.1.617.2 [Delta]	Negative

The Ion AmpliSeq technology and the Ion Torrent personal genome machine were used to sequence the genome of SARS-CoV-2 (PGM) (19). The data obtained was evaluated using the entire genome of the SARS-CoV-2 Wuhan-Hu-1 isolate after sequencing (GenBank accession number MN908947.3). Five specimens with >90% coverage yielded consensus of the whole genome data of SARS-CoV-2 and the remaining five were with partial genomes (mean genome length: 60.5%; Table 1). During dominance of the B.1.617.2 (Delta) and B.1.617.3 strains in the second wave of the COVID-19 pandemic in India (19), seven patients were infected with B.1.617.2 (Delta) strain, while the other two were infected with Kappa and one strain remained unassigned (Table 1). The entire sequence database has submitted in EpiFlu of GISAID [https://www.gisaid.org/epiflu-applications/submitting-data-to-epiflutm/] global database [EPI_ISL_13964608-13964611].

The successful isolation of multiple strains of SARS-CoV-2 has been performed using Vero CCL-81 cell lines at Indian Council of Medical Research- National Institute of Virology (ICMR-NIV), Pune City, Maharashtra state, India (20). We attempted isolation of SARS-CoV-2 from fecal specimens in Vero CCL-81 cell lines (21). Similarly, attempts were made to infect Syrian Hamsters using protocols reported earlier (22). However, in spite of multiple efforts, isolation attempts were not successful. It has been documented using cell culture and animal models that infectious viral particles were not recovered from feces at the peak of infection, despite the infectious virus being recovered from respiratory specimens (23–26). Isolation of viable SARS-CoV-2 from a stool sample of a COVID-19 patient has been documented about 15 days after onset of disease (27). The failure to isolate SARS-CoV-2 using cell culture or animal models may also be due to very low viral concentrations. It has been documented that specimens containing <10^6^ copies per ml never yielded an isolate (23). It should be noted that all the specimens were collected before the 6th post-onset day in the present study and viral load was <10^4^ copies per ml.

The failure to isolate the virus has also been attributed to factors such as sampling bias, presence of inhibitor, and/or cytotoxicity of fecal specimens, etc. In future, fecal specimens spiked with virulent SARS-CoV-2 viral particles for attempts to re-isolate using cell culture and animal models needs to be done to confirm the same.

The transmission electron microscopic (TEM) analysis of the fecal specimens was carried out as described earlier (28). Samples were prefixed with one percent glutaraldehyde and negative stained, and the grid was examined under 100 KV in a TEM (Tecnai 12 BioTwin^TM^; FEI, The Netherlands). Images were captured using a side-mounted 2 k × 2 k CCD camera (Megaview III, Olympus, Japan). TEM imaging of clarified and negatively stained feces specimens (*n* = 10) showed the presence of distinct Coronavirus particles in two specimens (Figure 1). The size of the virus particle was 55 nm and the spike peplomer projections were ~20 nm in length. The frequency of detection of morphologically identifiable coronavirus particles was, however, quite low. In most of the samples, phage particles and bacterial debris in the form of disintegrated flagella and capsular material were observed. Wang et al. documented isolation of SARS-CoV-2 from stool specimens of four patients and observed live virus in two specimens using electron microscopy (29). It should be noted that SARS-CoV-2 and other Coronaviruses are indistinguishable morphologically from each other and occurrence of other Coronaviruses in the fecal specimens needs to be ruled out. Therefore, all the specimens were tested using RT-PCR assays consisting of specific primers and probes for detection of other coronaviruses namely HCoV-229E, HCoV-OC43, and HCoV-NL63 (30, 31). The RT-PCR assay, a highly sensitive technique, showed negative results indicating the absence of any other most common human coronaviruses in the fecal specimens of COVID-19 patients. In future, immune electron microscopic analysis needs to be done on TEM positive fecal specimens for further confirmation.

**Figure 1:**
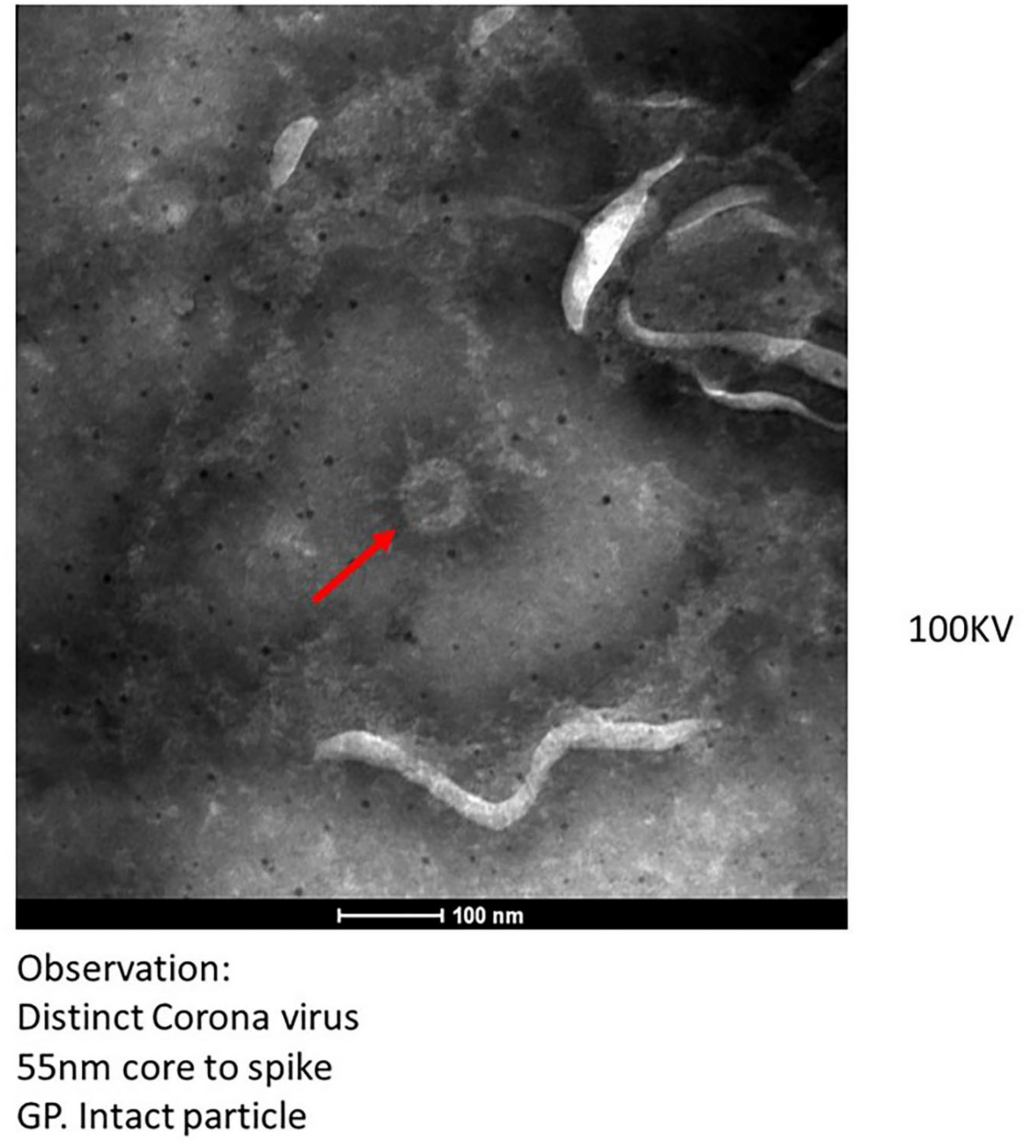
Transmission electron microscopy imaging from fecal specimen of positive COVID-19 patients.

The studies on presence of intact and infectious virion in fecal specimens are very scant. The electron microscopic technique used in the present study to visualize the intact coronavirus-like particles is a very time-consuming, labor-intensive process and requires a high level of skilled individuals. Similarly, for animal immunization and cell culture studies, handling of multiple specimens at a time is not possible and not recommended due to the possibility of cross contamination as the virus is replicating during passaging. Therefore, only 10 fecal specimens of COVID-19 patients were tested in the present study.

Even though several reports from different countries on the presence and persistence of SARS-CoV-2 in fecal/wastewater/sewage specimens are documented, a link is yet to be established between the spread of the infective virus from fecal specimens to the community. In this study, successful amplification of the full genome/nearly full genome and identification of the complete virion morphologically using TEM analysis in two specimens indicate the fecal excretion of the complete virion. However, the viability of the SARS-CoV-2 virion in fecal specimens was not confirmed using cell culture and animal studies. Future studies on a large number of fecal samples with high viral load as well as with late post-onset days needs to be done to explore the possibility of infection due to SARS-CoV-2 and to know its natural environmental persistence and infectivity in community settings.

## Data availability statement

The original contributions presented in the study are publicly available. This data can be found here: https://www.gisaid.org/epiflu-applications/submitting-data-to-epiflutm/EPI_ISL_13964608-13964611.

## Ethics statement

The studies involving human participants were reviewed and approved by ICMR-National Institute of Virology Ethical Committee. The patients/participants provided their oral/written informed consent to participate in this study.

## Author contributions

ML contributed in the conception of the work, experimentation was done by MJ, MS, and NC. SM contributed in the isolation of SARS-CoV-2 from fecal specimens. SP did the TEM experimentation work and analysis. Manuscript writing done by MJ and ML. All authors contributed to the article and approved the submitted version.

## Funding

This study was funded by the ICMR-National Institute of Virology, Pune, Maharashtra, India and Indian Council of Medical Research, New Delhi, India.

## Conflict of interest

The authors declare that the research was conducted in the absence of any commercial or financial relationships that could be construed as a potential conflict of interest.

## Publisher's note

All claims expressed in this article are solely those of the authors and do not necessarily represent those of their affiliated organizations, or those of the publisher, the editors and the reviewers. Any product that may be evaluated in this article, or claim that may be made by its manufacturer, is not guaranteed or endorsed by the publisher.
