# Supplementary material for: Lack of evidence of viability and infectivity of SARS-CoV-2 in the fecal specimens of COVID-19 patients
Source: Front Public Health. 2022 Oct 20;10:1030249. doi: 10.3389/fpubh.2022.1030249 (PMC9632423; doi:10.3389/fpubh.2022.1030249)
Supplement: Supplementary file 1 [file Table_1.DOCX]

**Supplementary**

**Methods**

**Collection of samples**

Fecal samples were collected from a laboratory confirmed COVID-19 patients (throat swab/nasal swab). Fecal samples were collected in the sterile container. All the fecal samples collected from the hospitals, transported to ICMR-National Institute of Virology, Pune and stored at -20°C until SARS-CoV-2 testing. To avoid contamination, all types of samples must be transported in sterile containers. While sample collection, workers should wear protective equipment, including disposable gloves, N-95 mask, solid front or wrap‐around gowns with sleeves that fully cover the forearms, head coverings, shoe covers, and face shield.

**Extraction of RNA from fecal specimens**

Approximately 30% fecal suspensions in 0.01 M phosphate buffered saline (PBS), pH 7.4 were prepared by centrifuging the suspensions at 4000 rpm (Hettich Universal 320R centrifuge) for 10 min to remove the debris. The viral RNA was extracted from 30% (w/v) suspensions of fecal specimens using spin columns Qiagen Viral RNA extraction Kit (Qiagen, Hilden, Germany) as per manufacturer's instructions.

**Real-time reverse-transcription polymerase chain reaction and determination of the copy number**

Detection of SARS‐CoV‐2 viral RNA (which genes) from the fecal specimen was done in accordance with the protocol described earlier (Chaudhary et al 2020). A 25-μl reaction was set up containing 10 μl of RNA extracted from fecal samples,12.5 μl of 2 X reaction buffer provided with the Superscript III one step RT-PCR system with Platinum Taq Polymerase (Invitrogen), 0.5 μl of reverse transcriptase/Taq mixture from the kit and 1.5 μl of primer probe mix for each reaction.

Thermal cycling was performed at55°C for 10 min for reverse transcription, followed by 95°C for 3 min and then 45 cycles of 95°C for 15 s, 58°C for 30 s.

A standard curve of RNA transcript generated from SARS-CoV-2 of known titer was used to quantify viral load (Choudhary et al 2020) and the number of viral genome equivalent copies was calculated (E and ORF 1b gene: 10^6^ copy/μL, with 29 and 28Ct respectively).

**Isolation of SARS CoV-2 from fecal specimens**

Attempts of SARS CoV-2 isolation in Vero CCL-81 cell lines were carried out as per earlier published method (Sarkale et al 2020). Along with this i*n-vivo* virus isolation approach was also attempted in Syrian hamsters (Yadav et al 2022). The Syrian hamsters (8–10 weeks old) were anesthetized using the Isoflurane. A hundred microlitre volumes of each specimen were inoculated through the intranasal route into two hamsters. The nasal turbinate (NT), lung specimens of passage 1 (P-1) were further passaged into new batch of hamsters (P-2). The hamsters were sacrificed and NT, lung specimens were collected on the 3rd day post-infection (DPI). NT and lung tissues (20% suspension) homogenates were prepared in sterile Minimum Essential Medium (MEM; Gibco, Waltham, MA, USA) using a homogenizer and were screened using qRT-PCR. SARS-CoV-2 positive NT and lung homogenized suspension of hamsters (P-1 and P-2) were further inoculated onto Vero CCL-81 cells.

The virus isolation experiment was conducted in a biosafety Level-4 facility at our institute.

**Whole genome sequencing**

Genome sequencing of SARS-CoV-2 was performed on the Ion AmpliSeq technology and the Ion Torrent personal genome machine (PGM).cDNA was synthesized with the SuperScript VILO reverse transcriptase kit (Invitrogen, USA) and the libraries were prepared according to the manufacturer’s instructions(Potdar et al 2020).

After sequencing, data was analyzed with the complete genome of the SARS-CoV-2 Wuhan-Hu-1 isolate (GenBank accession number MN908947.3) using programs Bowtie 2 version 2.3.3.1 and Burrows-Wheeler Aligner (BWA) version bwa-0.5.9 programs. All the sequences were submitted in GISAID database.
