# Supplementary figures and images for: Lack of evidence of viability and infectivity of SARS-CoV-2 in the fecal specimens of COVID-19 patients
Source: Front Public Health. 2022 Oct 20;10:1030249. doi: 10.3389/fpubh.2022.1030249 (PMC9632423; doi:10.3389/fpubh.2022.1030249)

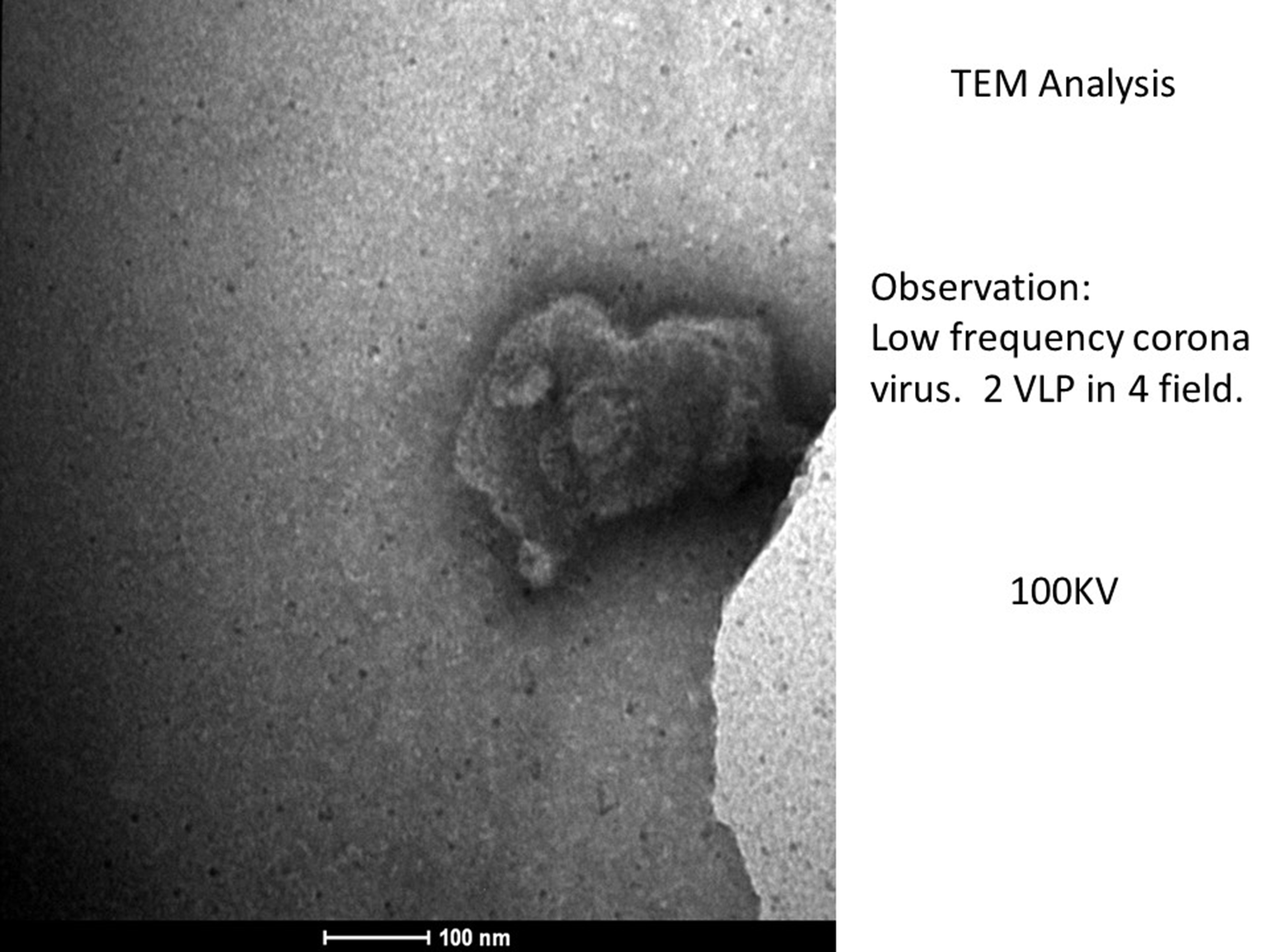

Supplement: Supplementary file 2 [file Image_1.JPEG]
